# A review on inertial microfluidic fabrication methods

**DOI:** 10.1063/5.0163970

**Published:** 2023-10-19

**Authors:** Zohreh Akbari, Mohammad Amin Raoufi, Sheyda Mirjalali, Behrouz Aghajanloo

**Affiliations:** 1 Department of Mechanical Engineering, Ferdowsi University of Mashhad, Mashhad, Iran; 2 School of Engineering, Macquarie University, Sydney, New South Wales, Australia

## Abstract

In recent decades, there has been significant interest in inertial microfluidics due to its high throughput, ease of fabrication, and no need for external forces. The focusing efficiency of inertial microfluidic systems relies entirely on the geometrical features of microchannels because hydrodynamic forces (inertial lift forces and Dean drag forces) are the main driving forces in inertial microfluidic devices. In the past few years, novel microchannel structures have been propounded to improve particle manipulation efficiency. However, the fabrication of these unconventional structures has remained a serious challenge. Although researchers have pushed forward the frontiers of microfabrication technologies, the fabrication techniques employed for inertial microfluidics have not been discussed comprehensively. This review introduces the microfabrication approaches used for creating inertial microchannels, including photolithography, xurography, laser cutting, micromachining, microwire technique, etching, hot embossing, 3D printing, and injection molding. The advantages and disadvantages of these methods have also been discussed. Then, the techniques are reviewed regarding resolution, structures, cost, and materials. This review provides a thorough insight into the manufacturing methods of inertial microchannels, which could be helpful for future studies to improve the harvesting yield and resolution by choosing a proper fabrication technique.

## I. INTRODUCTION

Among passive microfluidic approaches for particle manipulations, inertial microfluidic has been considered a favorable method for filtration and separation owing to its simplicity, ease of fabrication, and high throughput.^1–3^ While a majority of microfluidic systems function in low Reynolds numbers (Stokes regime), inertial systems operate in an intermediate range of Reynolds number (Re), between Stokes and turbulent regime, resulting in significantly higher throughput.^4–9^

The mechanism of inertial focusing depends on the balance between inertial lift forces and viscous drag forces.^5,10–12^ Inertial lift forces can be mainly categorized into two distinct groups: wall-induced lift forces arising from the interaction between suspended particles and the adjacent wall, and shear-gradient lift forces stemming from the parabolic curvature exhibited by the velocity profile. Shear-gradient forces direct particles toward the walls, while wall lift forces push particles away from the walls. Previous studies proved that the blockage ratio, which is defined as the ratio of the particle's diameter to the hydraulic diameter of the channel, must surpass the threshold of 0.07 for effective particle focusing.^13^ As the blockage ratio decreases, the effectiveness of particle focusing deteriorates.^14,15^ Therefore, the precision of channel dimensions plays a crucial role in achieving effective particle focusing.

Different configurations for inertial microfluidic devices have been recognized, including straight channels,^16,17^ contraction–expansion channels,^18,19^ and curved channels.^20–22^ Overall, the number of particle focusing bands can be modified by the changes in the cross section and the geometry of the microchannel.^23^ In square channels with an aspect ratio (AR) = 1, four equilibrium positions have been observed near the center of the channel edges.^10,11^ Replacing the cross section with a rectangular (AR ≪ 1) reduces the number of equilibrium positions into two lines.^12^ Modifying the cross section from square or rectangular to trapezoidal leads to alterations in the velocity profile within the channel, consequently influencing shear-gradient lift forces. The asymmetry in the velocity profile within the trapezoidal cross section results in the displacement of the center of maximum velocity toward the longer side of the trapezoid, thereby directing particle focusing toward the longer side wall.^24^ Furthermore, when altering the cross section to a triangular shape, the number of particle focusing streams decreases to one; nonetheless, this outcome is significantly contingent on the channel's aspect ratio. For example, Kim *et al*.^25^ investigated two types of triangular microchannels (narrow and wide triangular channels) and observed three focusing bands. Mukherjee *et al*.^26^ also studied the effect of the low aspect ratio triangular cross section on particle focusing. They observed that single-stream particle focusing at the location of 40% of the channel height. Numerous numerical studies have also been conducted to illustrate the influence of the channel's geometry on particle focusing.^6,16,24^ Furthermore, more complex geometries such as spiral or contraction–expansion arrays will reduce the number of particle focusing positions and form a tight focusing band due to the secondary flow and Dean drag forces exerted on particles.^5,8^

Conventional inertial microfabrication methods include photolithography,^13,27^ micromachining,^24,28,29^ 3D printing,^19,30–33^ hot embossing,^34,35^ microwire,^36^ etching,^25,37^ and laser cutting.^20,38^ Each of these methods is well suited for specific geometries and purposes, owing to their unique characteristics such as resolution and surface quality. For instance, high surface roughness influences the velocity profile within the microdevice and increases turbulence effects. Therefore, in inertial microfluidics, high surface resolution is crucial since flow disturbance increases in higher turbulency.^19,29,39^

Apart from the geometry, material selection plays an important role in the microfabrication method due to the use of inertial microfluidics in medical and biological applications. One key factor in material selection is biocompatibility. For example, hydrophobic materials absorb drug and biological molecules or swell up in direct contact with the organic materials. Also, further factors such as transparency, availability, cost-effectiveness, and non-toxicity are important in material selection.^20,40,41^

In this study, for the first time, the conventional fabrication methods in inertial microfluidics have been reviewed. This review provides comprehensive information about parameters influencing the microchannel's finish quality to enhance the particle focusing at high throughput. First, the physics of inertial microfluidics and the common materials employed for manufacturing inertial microfluidic devices are briefly introduced. Then, inertial microfabrication methods with their pros and cons including surface quality, cost, and ability to fabricate unconventional cross sections are explained. Subsequently, these methods are compared based on different criteria, and finally, the prospects of inertial microfluidic devices and their innovations are described.

## II. PHYSICS OF INERTIAL MICROFLUIDICS

In inertial microfluidic systems, randomly dispersed particles are mainly affected by inertial lift and Dean drag forces.^28,42^ Inertial force includes shear-gradient and wall-induced lift forces in which shear-gradient lift force directs particles toward the walls while wall-induced force pushes the particles away from their adjacent walls.^43^ The net inertial lift force can be estimated as follows:^1^

$$F_{L}=\frac{\rho_{f}U_{m}^{2}a_{p}^{4}}{D_{h}^{2}}f_{L}(Re_{c},X_{p}),$$ (1)

where $\rho_{f}$, $U_{m}$, $a_{p}$, and $D_{h}$ are fluid density, the maximum velocity of the flowing fluid, particle diameter, and hydraulic diameter of the channel, respectively. $f_{L}(Re_{c},X_{p})$ also represents the coefficient of net inertial lift force, which is a function of normalized positions of particles along the channel cross section $\left(X_{p}\right)$ and Reynolds number.

Dean drag is another effective force acting on the particles in curved channels. This force forms by the pressure gradient across the radial direction, causing the flow to move from the center toward the outer wall and return circumferentially toward the inner wall.^31^ These, two counter-rotating vortices, known as Dean vortices, make particles rotate throughout the curved part. The Dean drag force (*F_D_*) can be estimated using Stokes' equation^20^ for 1 < *Re* < 100 as follows:

$$F_{D}=3\pi \mu U_{D}a_{p},$$ (2)

where $U_{D}$ and *a_p_* are the velocity of secondary flows and the particle diameter, respectively.

According to the equations, these driving forces are highly dependent on the channel geometry due to its effects on flow profiles, *Re* number, hydraulic diameter, and inertial force coefficient. The channel's rough surface or low resolution can disturb the laminar flow and decrease the focusing/separation efficiency. It also traps the small-sized particles and over time can block the channel. In addition, the local Re number is closely related to the shape of the channel.^44^ For instance, when a rectangular cross section alters with trapezoidal ones, the asymmetry of the trapezoid alters the velocity profiles, which creates stronger Dean vortices near the outer regions and enhances separation efficiency.^45,46^

Therefore, even a small change in the channel geometry can significantly change the focusing position and separation efficiency. However, precise fabrication of complex geometries is still a challenging issue in microscales. So, Secs. III–IV describe the current methods for the fabrication of inertial microfluidic systems and compare their cons and pros comprehensively.

## III. FABRICATION OF INERTIAL MICROFLUIDIC SYSTEMS

Enhancement in microfabrication methods and materials facilitates the construction of inertial microfluidic systems with complex cross sections. Primary materials used in microfabrication are silicon and glass due to their biocompatibility, availability, inertness, and excellent strength. These characteristics are essential for inertial microfluidic devices that operate at higher pressure compared to other microfluidic devices.^47–49^ However, advances in fabrication technologies and material science introduced different types of polymers suitable for microfabrication.^50,51^ This section compares the materials and microfabrication techniques used in inertial microfluidics.

### A. Materials

The earliest materials used as a substrate for the fabrication of microfluidic devices were silicon and glass due to their availability, compatibility, and excellent surface characterization.^52,53^ Silicon is expensive and optically opaque, which restricts its use for inertial applications where tracing fluorescent particle trajectories is crucial.^54^ Glass, on the other hand, has excellent, dimensional stability, chemical inertness, and the ability to bond to itself or polydimethylsiloxane (PDMS) by thermal bonding or oxygen plasma treatment.^20^ However, providing a high temperature, pressure, and a clean environment for the bonding process, increases the fabrication cost of glass-based microfluidic devices. Moreover, silicon and glass are not gas permeable, which hampers their application for long-term biological applications such as cell culture.^47^

Later on, organic polymeric materials with the advantages of low cost, nontoxic, biocompatibility, gas permeability, and optical transparency have been introduced to fabricate microfluidic devices.^50^ The fabrication cost of organic polymer-based microchannels such as PDMS is at least 10 times lower than glass. Overall, organic materials used for inertial microfluidic devices are categorized into elastomeric and plastic polymers.^55^ Elastomeric materials, for instance, PDMS are partially cross-linked polymers that can be stretched under external forces.^56,57^ PDMS-based microfabrication technique (soft lithography) has a great advantage of the facile fabrication process through mixing, casting on master mold, and curing of degassed PDMS to replicate microchannels with different structures.^58,59^ However, due to the elasticity of these materials, the PDMS-based microchannels would be deformed under high pressure, particularly for microchannels with a high aspect ratio.^60^

Furthermore, PDMS is auto-fluorescent at short wavelengths, which reduces the sensitivity of fluorescent detection in inertial microfluidic applications.^56^ Moreover, the intrinsic hydrophobicity of PDMS results in the absorption of some drugs, proteins, and hydrophobic molecules, which restricts its application in biomedical applications.^61^

Plastics are another type of material used for the microfabrication of inertial microfluidic devices. The characteristic properties of plastics are similar to elastomers except for their stiffness at room temperature, gas impermeability, and lower antifouling. Polymethylmethacrylate (PMMA), one of the most well-known plastic polymers, is widely used for the fabrication of microfluidic devices. It has an elastic modulus of 3.3 GPa, which shows more rigidity than elastomers such as PDMS with an elastic modulus of 300–500 kPa. In addition, PMMA is optically transparent from visible to UV wavelengths and could be easily machined at low temperatures (∼100 °C).^59^

With the development of fabrication techniques and the emergence of 3D printing methods, other types of materials used for 3D printing were developed. The most common 3D printing methods utilize either extrusion of heated material from a nozzle or curing photocurable resin to form the desired structure.^62^ The second technique employs an ultraviolet (UV) lamp or laser beam for irreversible cross-linking of polymers through curing printing material. Therefore, after the curing process, the cross-linked structures cannot be reshaped or melted, resulting in the fabrication of rigid and sometimes brittle shapes. It should be noted that these materials are costly, not transparent (yellowish color), and not biocompatible.^63^ In addition, the commercialized 3D printing materials are acrylonitrile butadiene styrene (ABS)-based, which absorb proteins and lipids molecules.^64^ However, 3D printable PDMS developed for stereolithographic 3D printing could bring new insights to benefit from both 3D printing and PDMS advantages.^65,67^

### B. Fabrication techniques

**FIG. 1.**
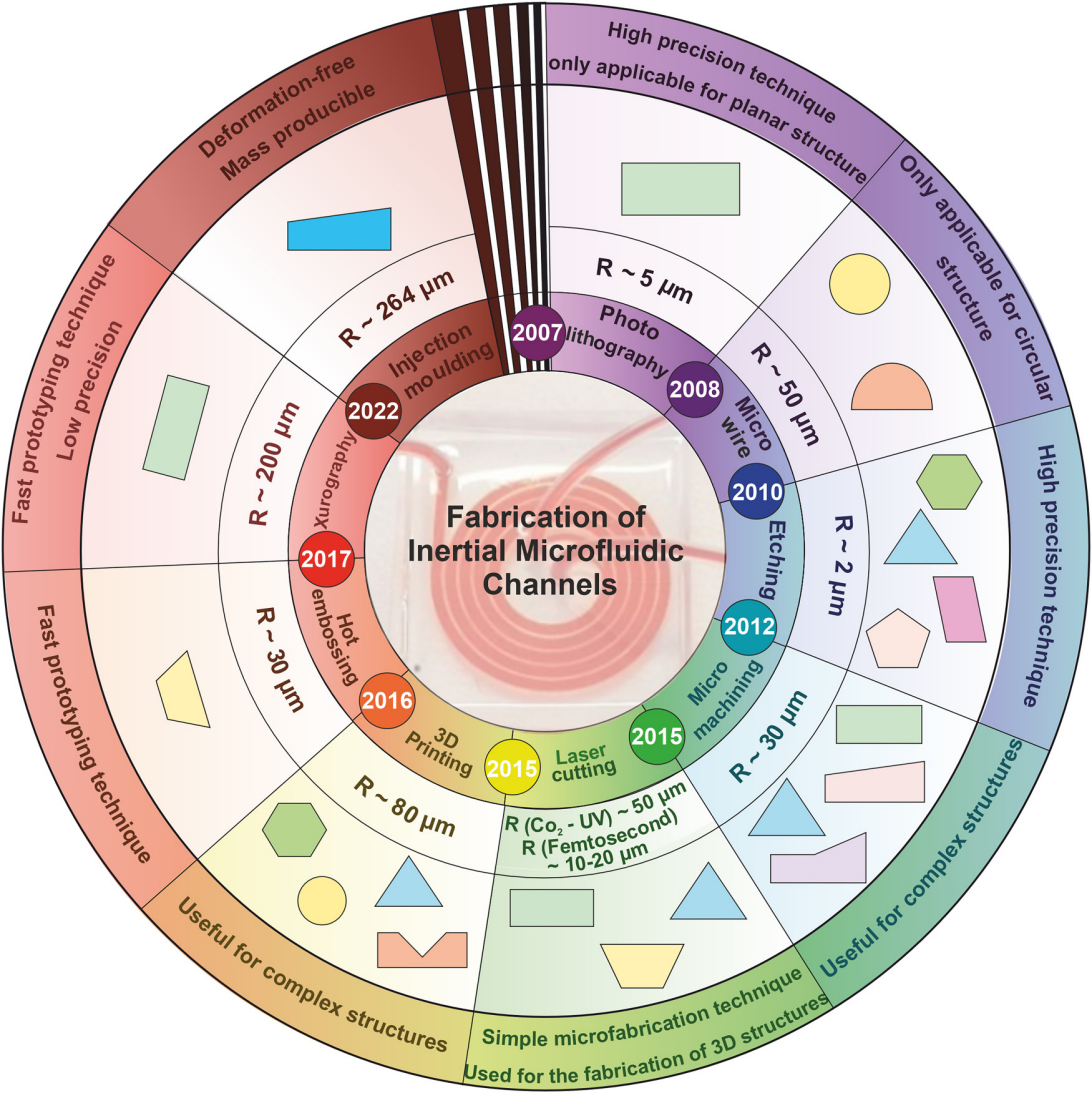
Timeline of developing inertial microfluidic fabrication methods.

Choosing an appropriate fabrication technique is important in manufacturing inertial microfluidic devices since the efficacy of the device critically depends on geometry, dimension, resolution, and cost, specifically in complex cross sections. Various techniques have been employed in the fabrication of microfluidic devices, but only a few of them are suitable for inertial microfluidic applications since most of the proposed methods cannot provide adequate surface quality, precision, and/or transparency. Inertial microfabrication techniques are typically categorized as photolithography, microwire, etching, micromachining, laser cutting, 3D printing, hot embossing, xurography, and injection molding. Primary studies in inertial microfluidics employed photolithography techniques for master mold fabrication. In 2007, for the first time, inertial migration and particle focusing behavior were investigated by Di Carlo.^67^ Microchannels were fabricated through a coating of SU-8 photoresist on a silicon wafer and defining the pattern photolithographically. However, due to some fabrication drawbacks in the photolithography method, later on, several attempts were made to develop new microfabrication techniques suitable for inertial applications. The historical timeline of these microfabrication techniques can be seen in Fig. 1. In Sec. III A 1, these techniques and their advantages and disadvantages are thoroughly explained.

#### 1. Photolithography.

**FIG. 2.**
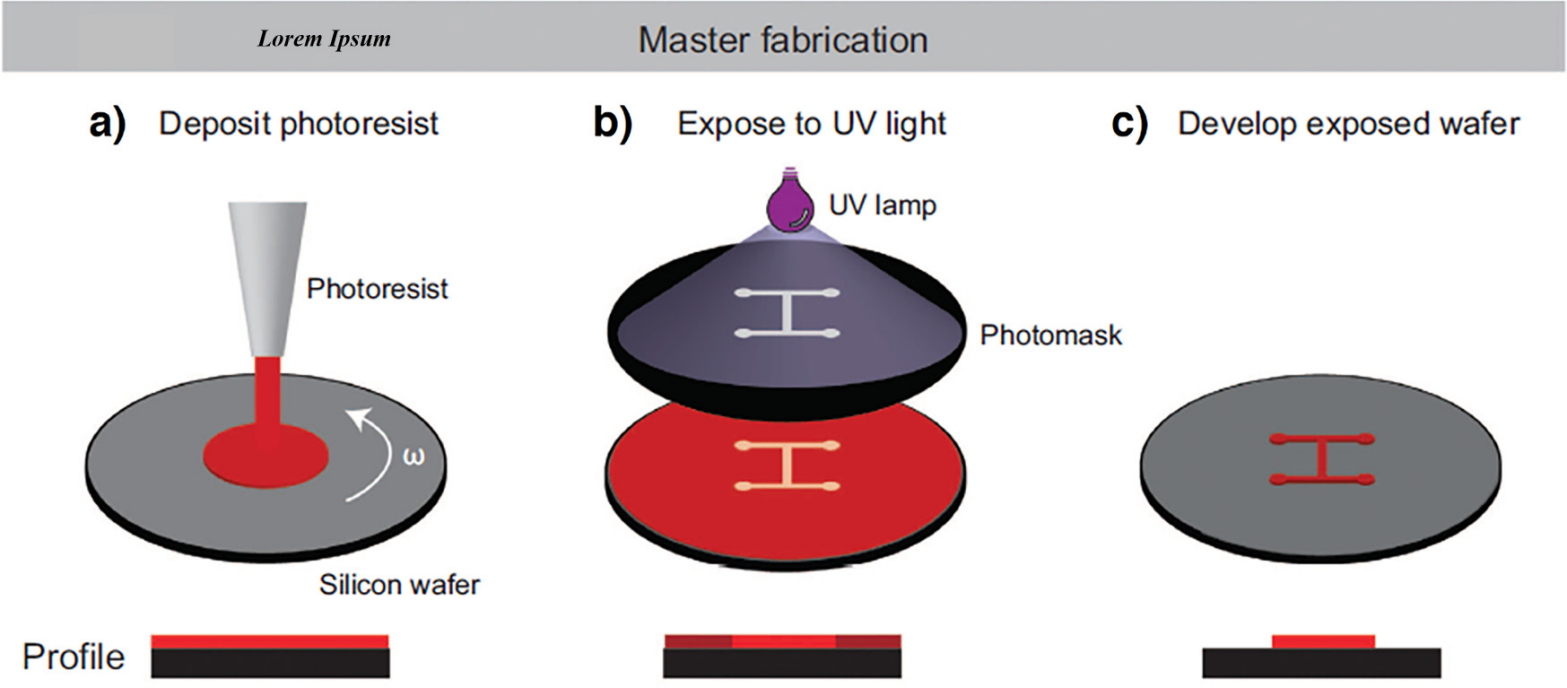
Schematic illustration of the mask fabrication by photolithography. (a) Depositing photoresist on a silicon wafer, followed by spin coating to make a uniform layer on the wafer. (b) Exposing the photoresist to the UV light through the mask. (c) Washing the non-exposed or exposed regions by the developer. Reproduced with permission from Aleklett *et al*., ISME J. **12**(2), 312–319 (2018). Copyright 2018 Nature Portfolio.

Photolithography, also known as optical lithography, is a process utilized to create patterns on a thin film. The thin film is often used as a master mold for slab replication.^68^ The schematic illustration of this process by negative photoresist is depicted in Fig. 2. This approach employs light to start the polymerization reactions and copy the patterns on the photoresist through photomasks. SU-8 was first developed in the 1990s and has been increasingly used for microfabrication due to the high resolution of the fabricated molds.^70^ This method can precisely produce extremely small structures, down to submicrometer sizes. However, the main drawbacks of this method are the expensive equipment, clean-room requirement, adroit operator, high time consumption, and inability to fabricate non-rectangular cross sections and non-planar geometries. Many devices have been fabricated for cell isolation, blood fractionation, cell separation, and filtration, using this method. However, all of them are limited to rectangular cross sections.

#### 2. Xurography

**FIG. 3.**
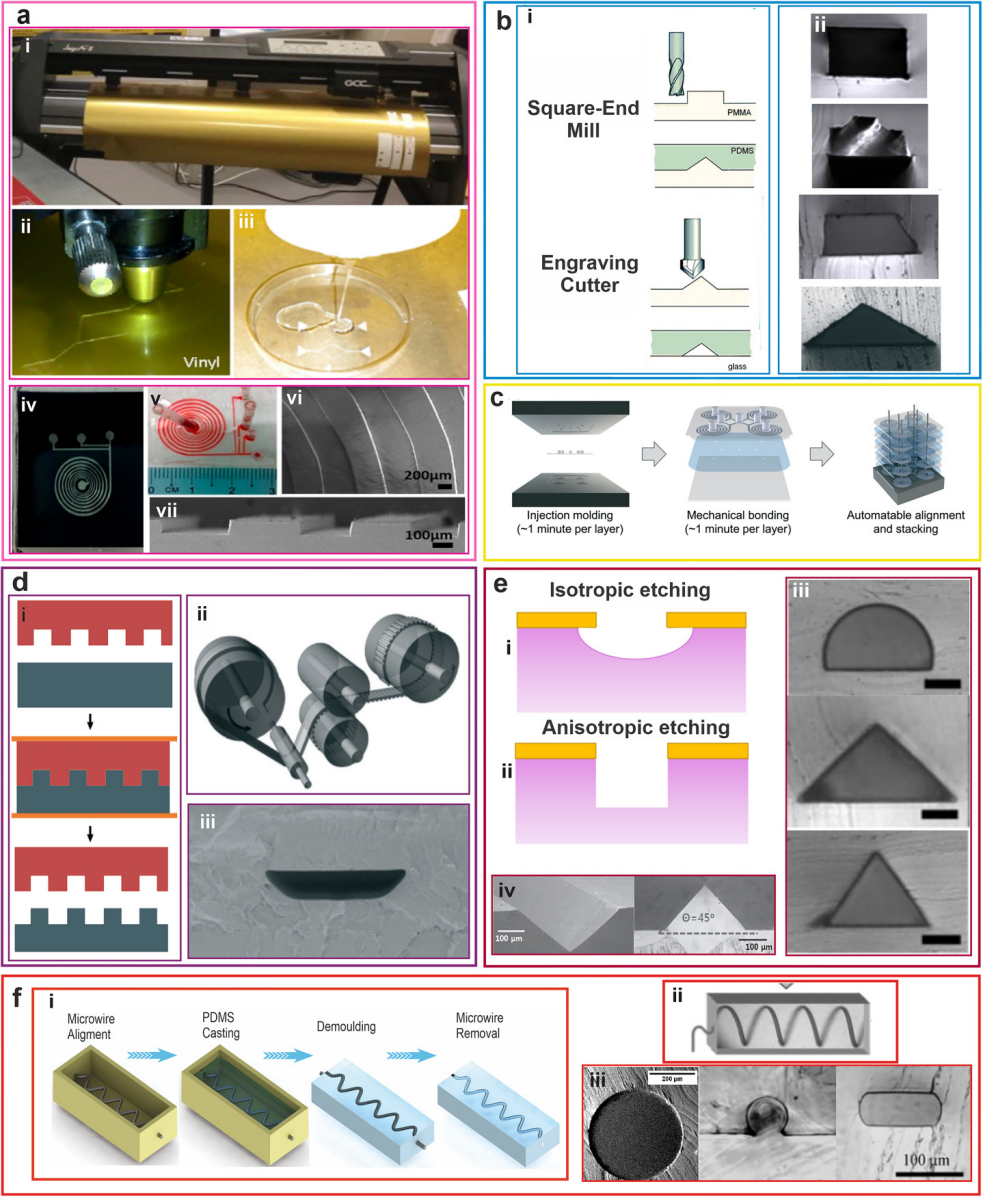
(a) Schematic illustration of xurography technique: (i) cutting plotter machine, (ii) cutting the vinyl film by cutting plotter to form the desired pattern, (iii) using adhesive film as a mold for PDMS molding and final microchannel fabrication [Reproduced with permission from Faustino *et al*., J. Biomech. **49**(11), 2280–2292 (2016). Copyright 2016 Elsevier], (iv) an epoxy master mold of spiral microchannel fabricated through xurography [Reproduced with permission from Mohamed Yousuff *et al*, AIP Advances **7**(8), 085004 (2017). Copyright 2017 AIP Publishing LLC]. (v) The final microchannel fabricated by PDMS and xurography. SEM images of (vi) top view, (vii) cross-sectional views of the microchannel [Reproduced with permission from Caffiyar *et al*., Micromachines **11**(5), 514 (2020). Copyright 2020 MDPI]. (b) Micro machining technique: (i) machining process [Reproduced with permission from Mukhrejee *et al*., Lab Chip **19**(1), 147–157 (2019). Copyright 2019 Royal Society of Chemistry], (ii) cross-sectional views of fabricated inertial microchannel via micro-milling [Reproduced with permission from Raoufi *et al*., Biomicrofluidics **13**(3), 034103 (2019). Copyright 2019 AIP Publishing LLC]. (c) Schematic diagram of a multiple stacked spiral microfluidic device based on injection molding method [Reproduced with permission from Jeon *et al*., Lab Chip **22**(2), 272–285 (2022). Copyright 2022 Royal Society of Chemistry]. (d) Hot-embossing method: (i) schematic illustration of the hot-embossing method, (ii) schematic of the roll-to-roll hot-embossing process, (iii) SEM image of the cross-sectional view of the fabricated microchannel [Reproduced with permission from Wang *et al*., Lab Chip **16**(10):1821–1830 (2016). Copyright 2016 Royal Society of Chemistry]. (e) Schematic illustration of the mold fabrication by (i) isotropic etching, (ii) anisotropic etching, (iii) SEM images of semi-circular, and triangular microchannel [Reproduced with permission from Kim *et al*., Lab Chip **16**(6), 992–1001 (2016).^25^ Copyright 2016 Royal Society of Chemistry], (iv) SEM image and optical microscopy of V-grooved silicon mold and PDMS cast after etching process [Reproduced with permission from Koh *et al*., Appl. Phys. Lett. **105**(11), 114103 (2014). Copyright 2014 AIP Publishing LLC]. (f) Micro wire technique: (i) schematic diagram of the micro wire process, (ii) images of the final fabricated microchannel [Reproduced with permission from Verma *et al*., Langmuir **22**(24), 10291–10295 (2006). Copyright 2006 ACS Publications], (iii) cross-sectional view of microchannel fabricated by micro wire technique. [Reproduced with permission from E. Effati and B. Pourabbas, Mater. Today Commun. **16**, 119–123 (2018). Copyright 2018 Elsevier and from Jia *et al*., Chin. Sci. Bull. **53**(24), 3928–3936 (2008). Copyright 2008 Springer.]

Xurography is a microfabrication technique in which a cutting plotter machine is used to remove the surplus materials from the adhesive vinyl film [Fig. 3(a)(i and ii)].^71^ This technique could be employed to directly fabricate microchannels or create masks and master molds without the necessity of clean-room and sophisticated facilities.^80^ In the case of master molds, the mold is covered by elastomeric polymers such as PDMS and then cured by microwaves [Fig. 3(a)(iii)]. Figure 3(a)(iv and v) shows the master mold and fabricated spiral microchannel by the xurography technique with seven loops and a rectangular cross section. Compared to other conventional fabrication methods such as photolithography, xurography is a fast-prototyping technique. Hence, by using pressure-sensitive substrates, the fabrication time can be reduced to a few minutes.^81^ However, the process of PDMS molding based on the xurography approach takes more time in the range of 1–24 h.^81^

The interesting advantage of xurography is the initial investment, as the cost of typical professional xurography equipment is approximately 4000 USD. However, in recent years, the investment cost for this microfabrication technique reduced by at least 50%.^82^ Nevertheless, this technique suffers from poor resolution, which restricts its application for microfabrication with a channel dimension of less than 200 *μ*m **[**Fig. 3(a)(vi and vii)].^72,83^

#### 3. Micromachining

Machining refers to any process that involves cutting a piece of raw material into a desired size and shape by an accurately controlled material-removal procedure known as subtractive manufacturing [Fig. 3(b)(i and ii)]. It is a manufacturing technique that employs a computer numerical controlled (CNC) system with a high-speed spindle and rotating cutting tool to remove bulk material from the surface of a workpiece.^72^ The shape, material, and size of cutting tools play a crucial role in the fabrication of complex features, such as rounded or sharp edges, which are desirable for inertial microfluidic applications.^84^ In general, two procedures have been widely used for inertial microfabrication: (1) fabrication of final microchannel by direct micro machining of PMMA sheet or (2) micro machining of aluminum block or PMMA sheet to create a master mold.^84,85^ The achievable resolution for manufacturing inertial microchannel employing micro-milling is less than 30 *μ*m due to the small size of the cutting edge.^86^ For a long time, micromachining has been only used to fabricate rectangular and trapezoidal cross sections for inertial microfluidic applications.^87–89^ However, recently, by using an engraving tool with a pointed tip, Mukherjee *et al*.^26^ were able to fabricate a straight triangular channel [Fig. 3(b)(i), triangular cross section]. Furthermore, in another study, Raoufi *et al*.^16^ fabricated straight microchannels with rectangular, trapezoidal, and complex cross sections to investigate the mechanism of particle focusing in elasto-inertial systems [Fig. 3(b)(ii)].

Injection molding is a highly automated and controlled process that relies on a master mold commonly prepared by micro-machining. It can produce parts with consistent quality from one batch to the next. This is important for applications where reliability and performance are critical. Also, it is a high-speed process that can produce large volumes of parts in a short amount of time. This makes it ideal for applications where large quantities of parts are needed. Jeon *et al*.^75^ developed a multiple-stacked inertial microfluidic setup using injection molding. The process involved fabricating two types of molds, one for the top layer with connection ports and the other for the bottom channel side, using micro-milling. The plastic material used for casting was polycarbonate resin. The double-sided adhesive film was used to seal the channel side of the plastic spiral device. To make the multiplexed spiral device, a plastic device with protruded connection ports was used for the top layer and those without were used for device stacking [Fig. 3(c)].

It should be noted that the micro-milled microchannel usually suffers from surface roughness issues (0.1 *μ*m < Ra < 0.5 *μ*m) caused by the size and speed of the cutting tool.^90–92^ It is noteworthy that hard materials, for instance, thermoplastics, should be used for micro-milling.^72^ Due to the deformations of elastomers, micro-milling of PDMS and other elastomers would be extensively challenging and prevent efficient material removal.^93^ Furthermore, micro-milling does not use thermal processing to facilitate material removal, affecting the surface quality of the micro-milled workpiece.^84^ Other types of material such as glass are not appropriate for micro-milling due to their brittleness.^84^ However, by optimizing milling parameters and choosing proper materials, the micro-milling technique is recognized as a rapid prototyping method with the advantages of low-cost and clean-room-independent.^84,86^

Furthermore, another approach for creating a master mold is to use a cutting tool to make a scratch on the surface of the mold by using a sharp cutting device made of a harder material.^94^ While creating straight triangular geometry with milling and scribing methods is simple, controlling the geometry and angles is very difficult, especially for non-rectangular geometries. In addition, the scribing approach not only generates a negative master mold, which rises a double-casting issue for the fabrication of the sealed microchannels but also limits the integration of other channels with these triangular channels.

#### 4. Hot embossing

Hot embossing technology is a flexible and low-cost fabrication method that involves precise stamping of a pattern in a polymer material softened by increasing its temperature just above the melting point [Fig. 3(d)(i)]. The sloped walls in the mold side facilitate de-molding the hot-embossed slabs.^95^ It uses glass or polymer substrates, which take an imprint of structures created on a master stamp. The resolution of this technique is in the order of 30 *μ*m. So far, various polymers have been hot-embossed successfully with micrometer/submicrometer-sized features, such as PMMA and polycarbonate.^96^ This method is mainly employed for patterning wells and microchannels for fluidic devices. The advantages of this method are the capability to function with a variety of thermoplastics, the ability for rapid prototyping, and high-volume production with microscale features. However, all the channels in this approach should be planar with orthogonal geometries, which limits the use of this method for the fabrication of non-conventional geometries. Few studies used hot embossing for the fabrication of rectangular microchannels suitable for inertial microfluidic applications. Wang *et al*. used the roll-to-roll hot-embossing method, as demonstrated schematically in Fig. 3(d)(ii), for the fabrication of an inertial microfluidic device with a rectangular cross section in PMMA.^35^ Despite the rectangular microchannel fabricated by soft lithography, the hot-embossed microchannel had a trapezoidal cross section [Fig. 3(d)(iii)].

#### 5. Etching

Etching involves the elimination of layers from the surface of a wafer by chemical reaction during the manufacturing process. There are two types of etching for microfabrication, including dry etching (plasma-phase) and wet etching (liquid-phase). The wet etching process is time-consuming, and controlling the edges and angles is troublesome in this method. Most of the etchants are isotropic and erode in all directions equally, which reduces the accuracy of the final structure [Fig. 3(e)(i)]. Inertial microfluidic devices require sharp edges and well-controlled features, which makes use of anisotropic etching a crucial need. Anisotropic wet etching (crystallographic etching) is more common due to its enhanced capabilities to erode in specific directions [Fig. 3(e)(ii)]. The anisotropic etchants etch crystalline materials (e.g., silicon) at very different rates according to the direction of the erosion and exposed crystal face. Among various anisotropic wet etchants available for silicon, potassium hydroxide (KOH) has been widely used for microfabrication.

In dry etching, plasma is used to generate active free radicals to react at the surface of the silicon wafer. It should be noted that as neutrally charged particles attack the silicon wafer from all directions, this process is classified as isotropic. To address this difficulty, reactive-ion etching (RIE) and deep reactive-ion etching (DRIE) were introduced to fabricate microfluidic systems accurately. Using this method, Kim *et al*.^25^ have constructed triangular channels with 90° and 70.6° tip angles to study the particle migration in non-rectangular cross-sectional geometries **[**Fig. 3(e)(iii)]. In addition, Koh *et al*.^37^ have used anisotropic etching of silicon mold to manufacture microchannels with V-shaped cross sections, as illustrated in Fig. 3(e)(iv). Although etching is capable of mold fabrication with dimensions down to the nanometer, the difficulty of controlling the geometry and angles limits its extensive use for creating master molds for inertial microfluidic applications. Also, anisotropic wet etching is typically limited to a few geometries as a result of crystallography.

#### 6. Embedding micro wire

Embedding micro wire is another approach for the fabrication of circular microchannels. In this method, a wire of the desired diameter is embedded in an uncured PDMS. When the PDMS is cured, the wire is gently pulled out of the PDMS block.^36,79^ The fabrication process of embedding micro wire is shown in Fig. 3(f)(i). Despite the simplicity of this approach for microchannel fabrication, this method is limited to the straight circular and semi-circular channels [Fig. 3(f)(ii and iii)], as wire removal in more complex channels can damage and rupture them. Apart from micro wire, there are other materials that can be used as an embedded mold; however, they have not been employed for microchannel fabrication due to the low quality of the replicated channels and the difficulty of mold removal from PDMS.^77–79^

#### 7. Laser cutting

**FIG. 4.**
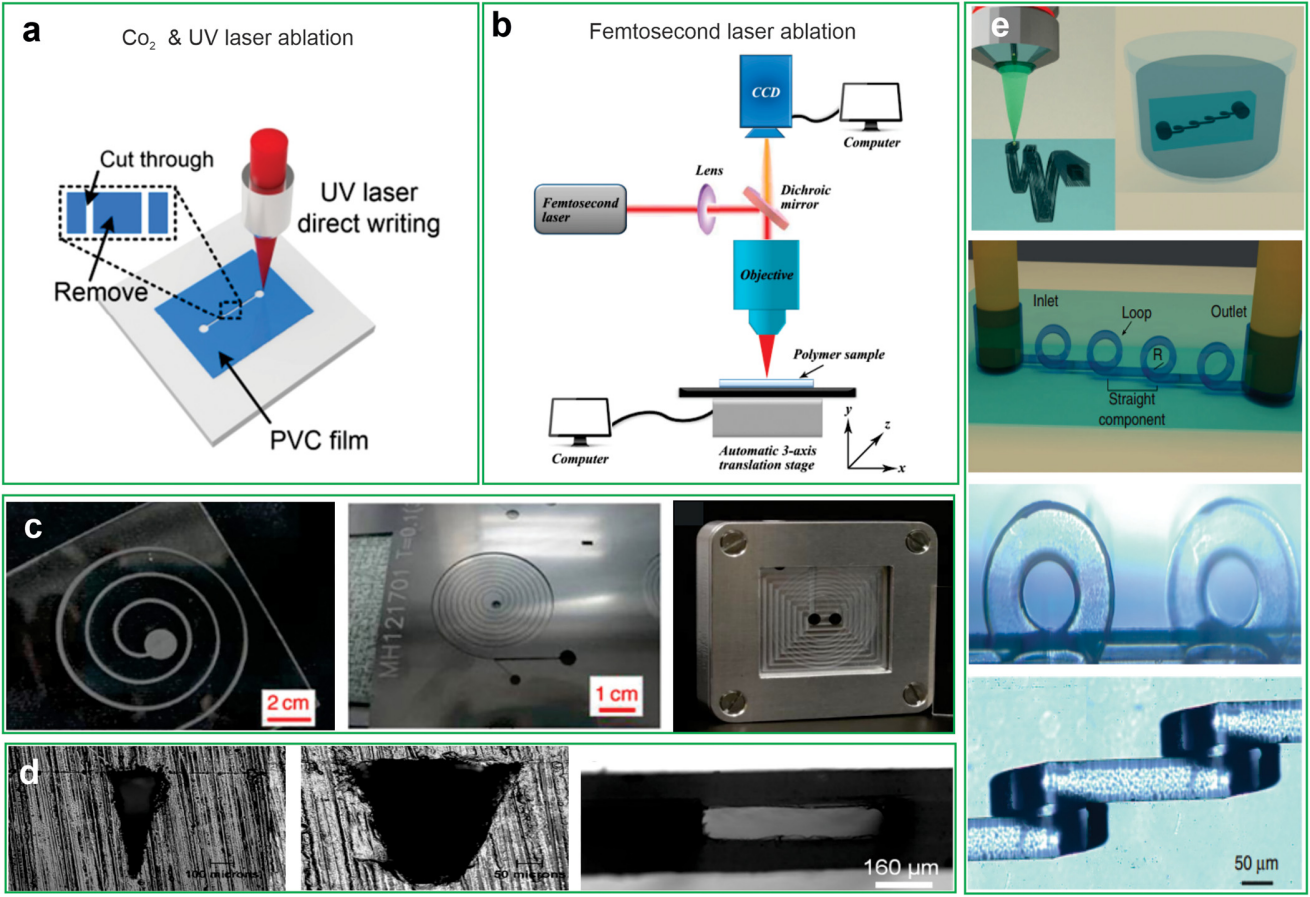
Schematic illustration of (a) CO_2_ and UV [Reproduced with permission from Zhang *et al*., RSC advances **6**(12), 9734–9742 (2016). Copyright 2016 Royal Society of Chemistry]. (b) Femtosecond laser ablation [Reproduced with permission from Wu *et al*., Mol. **23**(9): 2347 (2018). Copyright 2018 MDPI]. (c) Optical images of final fabricated microchannels and master mold [Reproduced with permission from Chung *et al*., Micro Nano Lett. **10**(10), 500–503 (2015). Copyright 2015 John Wiley and Sons and from Al-Halhouli *et al*., Micromachines **9**(4), 171 (2018). Copyright 2018 MDPI]. (d) SEM images of the triangular, trapezoidal, and high aspect ratio rectangular cross sections fabricated by CO_2_ laser ablation [Reproduced with permission from Su *et al.*, J. Renew. Sustain. Energy **12**(4), 046302 (2020). AIP Publishing LLC]. (e) Schematic diagram of manufacturing 3D inertial microchannel by femtosecond laser ablation technique [Reproduced with permission from Paiè *et al*., Microsyst. Nanoeng. **3**(1), 1–8. (2017).^99^ Copyright 2017 Springer].

Laser cutting is a rapid manufacturing method for the fabrication of inertial microfluidic devices that utilizes a laser plotter to engrave the surface of different materials.^72^ In this technique, the thermoplastic solid base is exposed to the laser beam to remove materials from the sheet surface. Generally, two different types of laser beams, long pulse [Fig. 4(a)] and short pulse [Fig. 4(b)] laser beams could be employed.^72^ The targeted pattern (final microchannel or master mold) is fabricated [Fig. 4(c)]. After ablation, the debris produced during the ablation process must be cleaned from the structure to prevent the microchannel from fouling.^70,100^ Due to the effect of heat concentration caused by long pulse laser beams (e.g., CO_2_ laser) on the surface of the workpiece, a minimum resolution of 50 *μ*m could be obtained through the CO_2_ laser ablation technique.^38,97,101^ In contrast, the minimum resolution could be reduced to 10–20 *μ*m by using short-pulsed UV and femtosecond lasers.^72,102^

The quality and type of achievable fabricated microstructure are highly dependent on the characteristic properties of the laser devices and the substrate. Some characteristic features of laser systems such as wavelength, the spot size of the beam, power, the pulse mode of the system, and absorption characteristics of the substrate, including melting and vaporization temperatures and absorption spectra of the workpiece, have a significant effect on resolution and final surface quality of the fabricated microchannel.^97,103^ The simplicity and fast prototyping of this technique are great advantages for inertial microfluidic applications. By optimizing laser parameters, the required time to fabricate inertial microchannel could be diminished (less than 1h), as well as a variety of cross sections such as trapezoidal and triangular could be obtained [Fig. 4(d)].^104^ However, this technique suffers from poor surface quality due to the resolidification, bulges, heat-affected zone during the laser process, particularly for CO_2_ laser machining.^38^ In addition, bulge formation at the edge of the microchannel during laser machining has a negative influence on the bonding process, leading to a lack of complete sealing and a higher risk of fluid leaks during operation.^105^ Several researchers have focused on improving the surface quality and reducing the heat-affected zone result in a metal foil-assisted technique.^38^

An alternative approach to minimize heat effects generated during laser processing is the implementation of femtosecond laser systems. In recent years, femtosecond laser processing has attracted tremendous attention due to its great advantages of ultra-short pulse width and high peak intensity, which minimize the generation of the heat-affected zone during laser processing and enable the fabrication of high-quality microchannels.^106^ The possibility of direct patterning of 3D microchannel structure inside a transparent material such as glass makes this technique a promising candidate for inertial microfluidic fabrication [Fig. 4(e)].^99^ Furthermore, due to the focusing femtosecond laser beam inside the glass, it is possible to fabricate a microchannel with no subsequent bonding to isolate the surface of the microchannel.^107^

#### 8. 3D printing

Additive manufacturing or 3D printing has emerged as a powerful platform for the fabrication of 2D and 3D structures with complex geometries. This technology can produce 3D objects with the material being added together (such as powder grains or liquid molecules), typically layer by layer. One of the key benefits of additive manufacturing is the capability to create complex structures or geometries readily while requiring only a digital 3D model or a computer-aided design (CAD) file. Based on the type of material and printing method, this technology can be classified into different groups. Among them, Stereolithography (SLA), Digital Light Processing (DLP), Fused Deposition Modeling (FDM), and multi-jet are the methods that have been widely used for microfluidic applications.^108^

##### Stereolithography

**FIG. 5.**
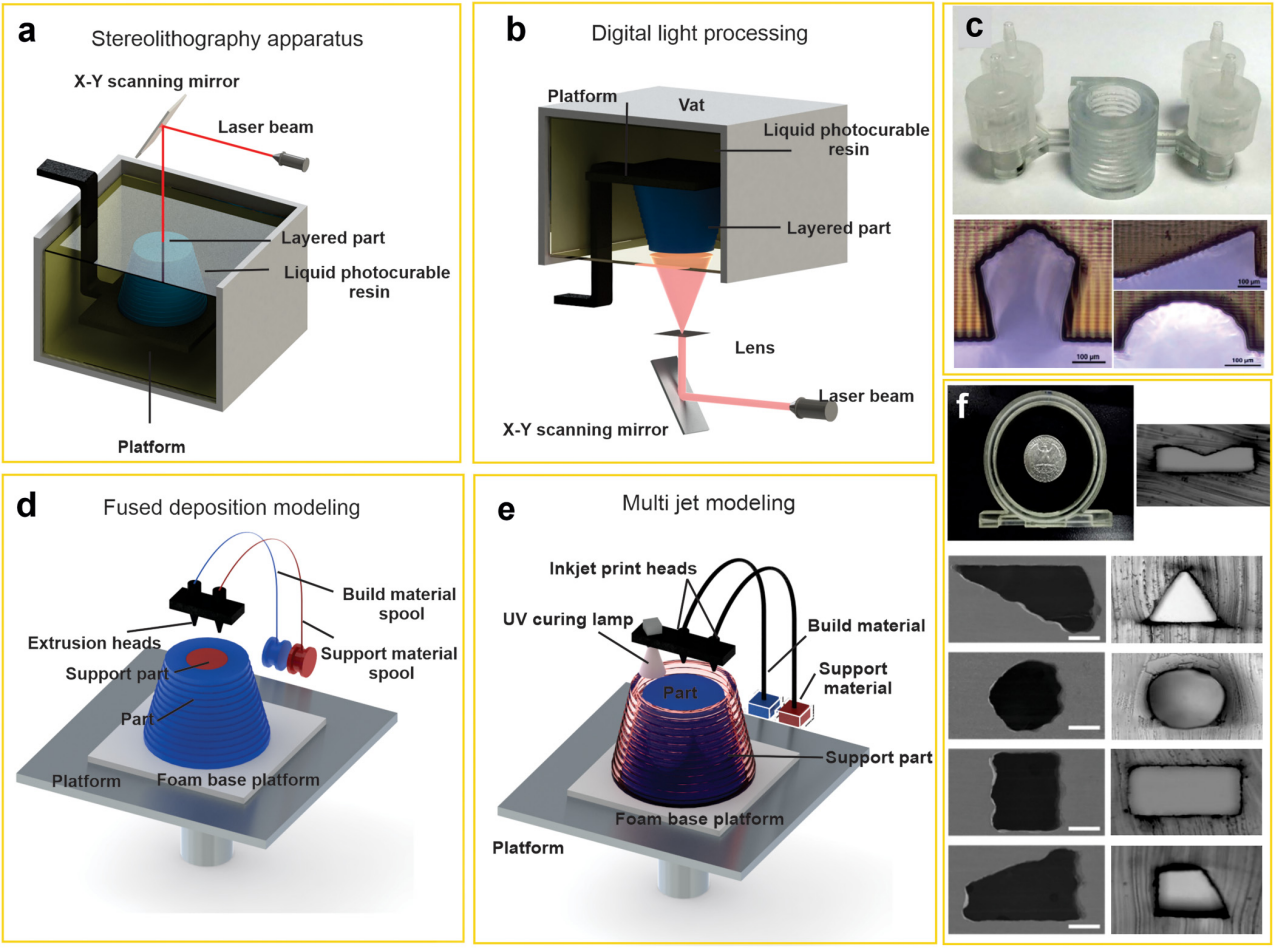
(a) Schematic illustration of SLA 3D printing technique, (b) DLP 3D printing technique, (c) optical images of SLA fabricated 3D helical microchannel and the cross-sectional view of fabricated microchannel by SLA/DLP technique [Reproduced with permission from Razavi Bazaz, Sci. Rep. **10**(1), 5929 (2020). Copyright 2020 Nature Portfolio and from Lee *et al*., Sci. Rep. **5**(1), 7717 (2015). Copyright 2015 Nature Portfolio], (d) FDM 3D printing technique, (e) multi-jet 3D printing technique, (f) images of fabricated microchannel by MJ 3D printing and their cross sections [Reproduced with permission from Chen *et al*., Sens. Actuators B Chem. **301**, 127125 (2019). Copyright 2019, Elsevier and from Raoufi *et al*., Soft Matter **16**(10), 2448–2459 (2020). Copyright 2020 Royal Society of Chemistry].

Stereolithography (SLA) has two important configurations: constrained surface approach (bat configuration) and free surface approach (bath configuration).^109^ In both configurations, structures are constructed layer by layer by controlling the photo-polymerization of a liquid resin spatially.^110^ In the bath design, a precise UV beam produces a planar geometry on a substrate immersed in a tank containing photoactive resin, which polymerizes under illumination [Fig. 5(a)]. After finishing each layer, the substrate moves further down into the resin, and the UV beam starts patterning the next layer on top of the former layer.^62^ Although SLA 3D printing systems have great benefits of the acceptable resolution, low cost, and reusability of remaining resin in a vat, the extensive cleaning procedures and chemical reactions with air in bath configuration are still the major concerns.^110,112^ However, in bat configuration, the object is constructed on a mobile substrate (picker) similar to a bat hanging from a ceiling [Fig. 5(b)]. The picker is suspended over the resin tank, and the UV light source is positioned below the reservoir.^63^

In contrast to traditional SLA systems, recently, the SLA 3D systems using visible light produced by a high-intensity laser beam or light-emitting diode (LED) beam have been considered. This process can be performed with either digital light processing or a scanning laser,^113^ which is called DLP 3D printing. In this procedure, unlike the conventional SLA systems, which cure the printed layer point by point, all the surface of the 3D printed layer is exposed to UV or visible light [Figs. 5(a) and 5(b)].^114^ The advantage of this method is the reduction of the printing time compared to traditional SLA 3D printing. In terms of resolution, the minimum feature size in the X–Y direction is determined by the pixel size of projection which is significantly smaller than laser spot size, resulting in a higher resolution of DLP-based 3D printing systems.^63^ The main difference between these two technologies is in the utilization of masks. The DLP method uses a digital process as a dynamic mask, while SLA is a mask-less process that uses a direct laser scanner [Fig. 5(c)].^115^

Despite the great benefits of SLA/DLP printers for the construction of directly printed parts,^111^ this method is not proper for creating microchannels due to the difficulty of residual removal from the printed microchannels. Moreover, the poor optical transparency of the current resins averts particle tracking for both fluorescent and non-fluorescent particles.^63^

##### Fused deposition modeling (FDM)

The fused deposition modeling (FDM) printer is one of the most broadly used 3D printing technologies. In an FDM machine, thermoplastic materials (in the form of filament) are extruded via a nozzle with high temperature to construct three-dimensional structures layer by layer [Fig. 5(d)].^116^ These plastics can be biocompatible and inexpensive.^117^ When one layer is printed, the build plate moves downward, allowing the extrusion nozzle to print another layer on top of the previously printed layer. In this method, unlike the previous technology, the extruded thermoplastics are solidified spontaneously after leaving the nozzle.^110^ Generally, microchannel fabrication using the FDM method has some major challenges, including low density, more prone to breakage, and low resolution (100 *μ*m) compared to other 3D printing techniques.^63,110^ In addition, the diameter of extruded thermoplastic (commonly 3–4 mm diameter) is bigger than the conventional size of a microchannel.^62,118^ However, recently, an FDM printer has been used to fabricate microchannels with non-rectangular geometries for inertial microfluidic application.^118^ The mold fabrication with this method is troublesome as the printer nozzle needs to be modified for each shape, and consequently, the final channels' surface quality is imperfect.

##### Multi-jet printing

Inkjet printing, or multi-jet printing, is a printer-based technique that utilizes multi-print heads to print digital image data on a substrate. The concept of this method is based on using photo resin or molten wax coming out from the print head to fabricate 3D structures [Fig. 5(e)].^110^

The print material and support material are extruded from the different print heads, layer by layer, to form a 3D structure and maintain the original shape of the 3D structure, respectively. Then, the printed object is cured by a UV lamp attached to the print head.^117^ After that, the build platform moves down to match the printing layer's height, and during an iterative process, printing the next layer is started. Since the final object is printed, the support material is eliminated by dissolving it into the solution or heating it.^119^ The advantages of this method include the ability to fabricate an object from multiple materials, fast processing time, high accuracy, and acceptable resolution. However, commercial printing materials are proprietary and costly, which leads to an increase in the final cost of a printed object, more than other 3D printing methods. Figure 5(f) demonstrates the optical image and cross-sectional views of inertial microfluidic devices fabricated by multi-jet modeling and 3D wax printing. Relying upon the resolution of the 3D printing device in x–y and z-directions, the final surface quality of microchannels varies.

## IV. TECHNICAL COMPARISON

The main objective in selecting the proper fabrication method is to meet the technical requirements and device functionality. In this section, previously introduced microfabrication techniques are compared from different points of view. The crucial factors for comparison include (1) resolution and microchannel dimensions, (2) capability to fabricate complex structures, (3) required equipment, facilities, and fabrication cost, and (4) the material compatibility of the fabrication method. Since the fabrication methods employed in the inertial microfluidic systems have different unique features, a comparison of the mentioned techniques could be very beneficial to highlight the strengths of each method specifically. Table I gives an overview of the advantages, disadvantages, and minimum resolution of reported fabrication techniques.

**TABLE I.** Comparison of the different fabrication methods.

Fabrication method	High resolution and high-precision method	Fast-prototyping technique	Fabrication of unconventional cross section	Requiring master mold	High surface quality	Simple fabrication technique	Effective cost-investment process	Mass production	Additional information
Photolithography	*								Disadvantages: Requiring clean-room environment Limited control over surface properties
Xurography		*				*			
Micromachining		*	*						Advantages: The ability to use directly without master mold fabrication Disadvantages: High surface roughness Possibility of tool breakage
Hot embossing	*	*		*				*	
Etching	*								Disadvantages: Require precise etching temperature control and mass transport control
Microwire technique						*			Disadvantages: PDMS rupture and distortion Challenges of residual removing
Laser cutting (CO_2_ and UV laser ablation)		*				*			Advantages:The ability to use directly without master mold fabrication Disadvantages: Poor surface quality require double-casting due to the fabrication of the negative master mold
Laser cutting (femtosecond laser ablation)									Advantages: The ability to use directly without master mold fabrication Not requiring bonding process
3D printing		*	*				*		Disadvantages: Poor transparency challenges of residual removing Poor surface quality especially for slanted geometries
Injection molding		*						*	Disadvantages: Poor transparency

### A. Resolution and minimum achievable dimensions

For many years, soft lithography has been the conventional fabrication process in academic research due to its high resolution and accuracy.^120^ However, this method requires sophisticated and expensive facilities, which hamper its widespread use.^121^ In addition, only planar and orthogonal features could be created due to the geometrical limitations of photolithography.^122^ Hence, other microfabrication methods have been developed to create more complex structures while maintaining the resolution to the desired range. Etching is a high-resolution fabrication approach for non-planar structures such as triangular, and complex cross sections with resolution in the range of a few micrometers.^25,37^ Nevertheless, due to the difficulty and requiring high-precision temperature control, this method could not replace soft lithography.^94^

Afterward, a lot of efforts have been made to develop low-cost techniques with an acceptable resolution for inertial microfluidic applications, which has resulted in the appearance of different types of fabrication methods including, xurography, laser cutting, mechanical machining, hot embossing, 3D printing, and injection molding. However, for high-precision applications, the resolution of the mentioned approaches does not match the achievable resolution of photolithography and etching.^62^ For example, xurography is a simple and fast prototype method for the fabrication of inertial microfluidic devices with different structures.^74,82^ Despite the simplicity and affordability of xurography, this method provides a resolution of less than 200 *μ*m, which is not adequate for the fabrication of complex 2D and 3D structures.^3,83^ CO_2_ and UV laser cutting and mechanical machining have better resolutions in the range of 50 and 30 *μ*m, respectively.^86,97^ The resolution of short-pulsed femtosecond laser processing is also in the order of 10–20 *μ*m, enabling the fabrication of small-size 3D structures, with the benefit of eliminating the bonding process. Both methods mentioned methods can create triangular and trapezoidal cross sections. Although both approaches can create sharp and rounded edges,^84^ there are more variations between the design and actual dimensions in laser-cutting due to the formation of a heat affection zone near the channel edge.^38^ The surface quality of the final part in micromachining may vary depending on the type and size of cutting tools, as well as operational parameters.^84^

Hot embossing is another promising method for the commercialization of inertial microfluidic systems. The surface quality of this method relies on the quality of the master molds and can be improved by polishing.^84^ It should be noted that only planar geometries can be obtained by this technique. Additive manufacturing technologies such as 3D printing have been introduced for the fabrication of complex inertial microfluidic systems, with a minimum resolution of 30 *μ*m.^31^ However, the smallest fabricated microchannel size is approximately 200 *μ*m.^62,64^ This problem arises from the impossibility of removing resin and supporting materials from small channels.^123^ In addition, due to the layer-by-layer printing procedure, the final microchannel suffers from poor surface quality. Injection molding can achieve high levels of precision and accuracy, allowing the fabrication of intricate and complex microchannels in dimensions down to around 50 micrometers with tight tolerances. This is because the process involves the use of a mold, which provides a highly controlled and repeatable environment for the molten material to flow into.^124^ However, complex designs with varying wall thicknesses or sharp corners are difficult to produce and may require secondary operations to achieve the desired shape. Also, surface imperfections on the final part, such as sink marks, flash, and warpage, are flaws in injection molding. Increasing wall thickness can lead to more shrinkage potential, while variations in thickness cause heat accumulation and differential shrinkage, affecting product integrity. Sharp corners also contribute to stress concentration and crack propagation, impacting structural performance. Predicting warpage, especially for parts with significant area-to-thickness ratios, is crucial for achieving flatness and quality. Addressing these issues can enhance product quality and production efficiency in injection molding processes.^125,126^

### B. Achievable structures

The geometry of inertial microfluidic devices has a direct impact on the direction and magnitude of inertial and Dean drag forces.^125^ Therefore, numerous efforts have been dedicated to employing a technique for the fabrication of complex structures.

The primary fabrication method used for inertial microfluidic systems was photolithography, which is only applicable for manufacturing planar and orthogonal cross sections, such as rectangular cross sections. The downside of this technique is the impossibility of making channels with negative sidewalls, fully enclosed channels, or overlapping channels. Hot embossing can fabricate master molds with trapezoidal cross sections. The slanted edge of the microchannel facilitates the de-molding of the final structure from the master mold.^35^ With the advances in microfabrication, manufacturing triangular cross sections have been enabled by anisotropic etching. However, this technique is restricted to a few geometries due to the addition of process complexity for some structures, such as spiral microchannels with unconventional cross sections.^25,37^ In addition, by optimizing the laser parameters, triangular and trapezoidal cross sections could be obtained through laser cutting.^105^

Micromachining, laser cutting, and 3D printing could provide microchannels with complex structures and different features. These techniques have been extensively examined as a promising method for fast prototyping and mold-free manufacturing of inertial microchannels with complex structures. In the case of micromachining, by utilizing appropriate tools, it is capable of constructing a wide range of features such as round or sharp edges.^84^ Rafeie *et al*.^127^ have designed and fabricated three innovative spiral microchannels with convex, concave, and ramp cross sections to add more inertial complexity and improve manipulation throughput.

More complex structures such as 3D helical structures could be fabricated by femtosecond laser processing.^99^ This technique has the unique advantages of eliminating the bonding process and the ability to produce the integrated structure inside the glass. However, the length of the directly fabricated micro inside the glass is limited to only 1 cm.^106,107^ In recent years, 3D printing has been explored extensively as an additive manufacturing method with the ability to fabricate different features based on user demands. It is a mold-free fabrication technique for manufacturing structures that are difficult to obtain by traditional fabrication techniques. For instance, by employing the wax 3D printing technique, Raoufi *et al*.^31^ have investigated particle focusing behavior inside the straight, curved, and spiral microchannels with circular, rectangular, triangular, and complex cross sections.

### C. Cost comparison

Fabrication expense is one of the main concerns in manufacturing inertial microfluidic devices, including setup, operation, and fabrication costs.^84^ Inertial microfluidic fabrication methods generally require sophisticated equipment and high-precision control systems, which restricts their applications for industrial and large-scale manufacturing. Therefore, in the selection of proper microfabrication techniques, it is essential to reach an optimum point between cost, resolution, and production volume. From the cost perspective, the cost of equipment, installation, operation, and maintenance should be considered. For example, photolithography, which is only used for academic and laboratory studies, is recognized as an expensive fabrication technique due to the necessity of clean-room facilities and expert operators.^72^

In contrast, micromachining has a lower setup cost compared to photolithography based on no need for clean-room facilities. While micromachining is more cost-efficient compared to photolithography, it has lower resolution. It should be noted that the final surface quality and resolution of the fabricated device highly depend on the investment cost for micromachining. For instance, the final cost of fabricated devices changes with the size and features of cutting tools, and manual or automatic control of the machine. In addition, the maintenance cost of micromachining, including periodical replacement of cutting tool due to breakage or wear has an indirect impact on the final production cost per chip of inertial microfluidic device. However, considering all these expenses, the final cost of this method is still remarkably lower than that of photolithography.^84^

Hot embossing is also a cost-efficient, automated manufacturing approach for mass production. The processing time of this technique is less than 1 s per workpiece.^35^ Therefore, hot embossing is the most cost-effective method among all introduced techniques for large-scale production. Although the investment cost of laser cutting and 3D printing is much lower than hot embossing, they are not suitable for mass-production purposes. It is worth noting that the fabrication of 3D complex structures using femtosecond laser cutting is time-consuming, labor-intensive, and expensive. At the present time, the investment cost of high-resolution 3D printers is relatively high and its throughput is low, which limits its use for nonacademic applications.^64^ Injection molding is also reasonable when the channel design is simple and mass production is required. Otherwise, the initial cost of launching its infrastructure is expensive.^75^

### D. Material comparison

Since inertial microfluidics is mostly used for biological and medical applications, material biocompatibility is a crucial factor in selecting appropriate fabrication methods. PDMS as the most widely used material in almost all inertial microfluidic devices is intrinsically transparent, porous, and hydrophobic.^128^ However, it would swell up in direct contact with some organic materials, which results in changing the channel dimensions and structures.^40^ Furthermore, the hydrophobic nature of PDMS-based microchannels can cause the adsorption of some drugs and small hydrophobic molecules by the surface of channels.^20,41^ With the advance of microfabrication techniques, other materials, such as polymers and thermoplastics, have emerged. Although the material properties are important for biological applications, each fabrication technique has its own material limitations. For example, thermoplastic materials are suitable for manufacturing microchannels by hot embossing and direct micromachining methods.^84^ PMMA is an excellent candidate widely used for microchip fabrication using these two methods owing to its thermoplasticity and rigidity.^54^ In contrast, glass is not a good candidate for micromachining due to its brittleness.^84^ However, glass is the only practical material for the fabrication of inertial microchannel by femtosecond laser ablation.^106^

Long-pulsed laser ablation techniques like UV and CO_2_ laser cutters can function with various materials such as PMMA, COP, and PS. Although these materials have a transparent nature, PS and COP may lose their transparency during laser exposure, restricting their application for research where observing particle trajectories is a crucial need.^129,130^ 3D printers also can work with different resins and polymers based on their model and fabrication process. However, the biocompatibility of 3D printing resins and polymers has remained a challenge. Furthermore, most resins used for SLA and DLP have poor transparency, which eliminates their use for inertial microfluidic studies. However, some transparent resins have recently been marketed, such as Watershed, Visijet SL Clear, and BV-003, which have been employed for the fabrication of inertial microfluidic devices. It is worth noting that Watershed resin may lose its transparency and become yellowish after a long time.^62^ Injection molding can work with a wide range of materials, including thermoplastics, thermosets, and elastomers. This allows the creation of parts with different physical properties, such as flexibility, rigidity, and durability. One of the key benefits of injection molding for inertial microfluidics is the possibility of working with thermoplastic polycarbonate resin, which has excellent dimension stability. This allows large-scale fabrication of deformation-free structures that are well suited for real-world and industrial applications. However, some materials may require specialized processing techniques or may not flow easily through the mold, limiting the range of materials that can be used.^75^

Table II summarizes all the mentioned fabrication methods and materials used in inertial microfluidics as well as their corresponding channel shape, size, and resolution.

**TABLE II.** Inertial microfluidic devices fabricated by different microfabrication techniques.

Method	Mold materials	Channel materials	Channel shape	Cross-section	Dimension error range (DE)/resolution range (RR) (*μ*m)	Processing time/cost	Actual dimensions range (*μ*m)
Photolithography				Rectangular Square	RR: 5		
Photolithography	Si wafer	PDMS	Symmetric and asymmetric spirals^94,131–133^ Straight and/or curve^67,134^ Serpentine^133^	Rectangular	…	…	20–300
Xurography				Rectangular Square	RR: 200		
Xurography	Adhesive vinyl sheet Epoxy polymer	PDMS	Spiral^74^	Rectangular	…	…	100–600
Micromachining			Rectangular Square Trapezoidal Triangular complex	RR: 30			
Micro-milling	PMMA	PDMS	Spiral^45,135,136^ Straight^26^	Trapezoidal^45,135,136^ Triangular^26^ Rectangular^45,118^	…	…	40–300
Micro-milling	Aluminium	PDMS	Spiral^42,87,137,138^ Straight^16^	Trapezoidal^16,42,87,137,138^ Rectangular^138^ Complex^16,137^	DE: ∼3–10 *μ*m RR: x–y: 10 *μ*m z: 2 *μ*m	…	30–600
Diamond cutting	Brass	PDMS	Straight^94^	Triangular	…	…	20–76
Injection molding	Duralumin	Polycarbonate resin	Spiral^75^	Trapezoidal	…	∼10 min	50–500
Hot embossing				Rectangular Square	RR: 30		
Roll-to-roll hot embossing	PMMA	PMMA	Straight^35^	Trapezoidal	DE: 5–0 *μ*m	…	40–220
Etching				Rectangular Square Trapezoidal Triangular Complex	RR: 2		
Deep reactive-ion etching	Si wafer PDMS	PDMS	Spiral^136,139^	Rectangular	…	…	10–500
Anisotropic wet etching	Si wafer PDMS	Si wafer PDMS	Straight^25,37^	Parallelogram Rhombus Polygon (3–6) Half circular	…	…	30–116
Embedding microwire			Circular Semi-circular	RR:50			
Microwire	Steel microwire Nylon microwire	PDMS	Straight^36^	Circular	…	…	50–181
Laser cutting				Rectangular Square Trapezoidal Triangular	RR: 50 (CO_2_ and UV laser ablation) RR: 10–20 (femtosecond laser ablation)		
UV laser ablation	…	PVC film	Spiral^97,140^	Rectangular	DE: 20 *μ*m RR: 50 *μ*m	0.−1–1.5 ¢/chip ∼20 min	50–760
Femtosecond laser ablation	…	Glass wafer Fused silica glass^99^	Spiral^20^ Complex straight^99^	Trapezoidal^20^ Rectangular^99^	…	5 €/chip ∼3.5–6.5h	40–600
Foil-assisted CO_2_ laser ablation or irradiation	PMMA	PDMS	Spiral^34^	Rectangular	DE: 20–30 *μ*m	…	80–23
3D printing				Rectangular Square Trapezoidal Triangular Circular Semi-circular Complex	RR: 50		
DLP/SLA 3D printing	…	SLA/DLP resin^19^ Photopolymer^141^ DSM Somos watershed XC11122^111^ PDMS^142^	Straight^19,141^ Spiral^19,142^ Serpentine^19^ Curvilinear^19,141^ Contraction–expansion^19^ 3D helical^111^	Rectangular^19^ Trapezoidal^19,142^ Triangular^19,111^	RR: x–y: 25–56 z: 5–100	∼2–5h	80–1000
FDM 3D printing	ABS plastics^118^	PDMS^118^ ABS^141^ PLA^141^	Straight^118^	Semielliptical^118^ Triangular^118^ Circular^118^ Rectangular^118^	RR: x–y: 100 z: 100	…	100–1200
Wax 3D printing	Midas castable material	PDMS	Straight Curvilinear Spiral^31^	Circular Rectangular Triangular Complex	RR: x–y: 24 z: 6	∼6h	50–250
Multijet 3D printing		VisiJet M3 crystal material	Helical^30^	River meander-like Circular Rectangular Trapezoidal	…	…	300–1500
Inkjet 3D printing	…	UV curable plastic	Straight^141^ Curved Serpentine	Rectangular			

## V. CONCLUSION AND FUTURE OUTLOOK

Inertial microfluidics has emerged as a powerful platform for high throughput and efficient separation of particles and cells. Early studies in this field were limited to planar cross sections such as rectangular and square. Advances in microfabrication technology made it possible to obtain trapezoidal, triangular, and complex structures. Although different channels have been designed to increase the microdevice efficiency, straightforward fabrication of these channels for industrial purposes is still a major challenge. Therefore, the present study provides a comprehensive review of the inertial microfluidic fabrication methods and compares their features, advantages, and limitations.

Overall, although photolithography and etching enable the fabrication of 2D structures with resolution in the order of a few micrometers, both methods suffer from requiring sophisticated equipment and clean-room facilities, which increases the final production costs. Furthermore, only orthogonal features can be obtained through photolithography. For size-based sorting applications that require a channel with dimensions of a few micrometers, both methods still demonstrate superior advantages compared to other fabrication methods.

In contrast, xurography, microwire, and hot embossing are cost-effective but have lower resolution and are incapable of building channels with dimensions in the order of a few micrometers. For circular cross sections, microwire is a low-cost and simple method that could be employed. However, due to the limitation of this technique, it is only applicable to straight and 3D helical microchannels. For mass production of planar structures, hot embossing and injection molding are the best choices.

Femtosecond laser ablation, micromachining, and 3D printing are perfect candidates for the fabrication of complex non-planar structures. The femtosecond laser ablation method facilitates the fabrication of rectangular 3D structures inside glass without requiring a bonding process. For more complex structures, micromachining and 3D printing have attracted tremendous attention. While micromachining is only capable of creating master molds, 3D printing can build both master molds and microdevices with unconventional structures.

In terms of industrial manufacturing, injection molding can produce large volumes of parts quickly and consistently, with high precision and accuracy, and the ability to work with various materials. However, the process also comes with disadvantages, such as expensive mold production, limited design flexibility, and surface imperfections on the final part.

In summary, among all inertial microfabrication techniques, 3D printing is an affordable and rapid method for manufacturing complex master molds and microchannels. The main advantage of 3D printing is the ability to fabricate unconventional structures with arbitrary cross sections. However, each of these manufacturing processes has its own set of advantages and disadvantages, and the choice of process depends on factors such as the desired production volume, part complexity, material properties, and cost.

## Data Availability

The data that support the findings of this study are available within the article.
